# Nonreceptor Tyrosine Kinase c-Abl-Mediated PHB2 Phosphorylation Aggravates Mitophagy Disorder in Parkinson's Disease Model

**DOI:** 10.1155/2022/9233749

**Published:** 2022-11-09

**Authors:** Yongjiang Zhang, Jiannan Wu, Weina Jin, Mengmeng Shen, Shiyi Yin, Xiaoyi Lai, Hongxia Ma, Menghan Jiang, Dongxue Sun, Junqiang Yan

**Affiliations:** ^1^Neuromolecular Biology Laboratory, The First Affiliated Hospital, College of Clinical Medicine of Henan University of Science and Technology, Luoyang 471003, China; ^2^China National Clinical Research Center for Neurological Diseases, Jing-Jin Center for Neuroinflammation, Beijing Tiantan Hospital, Capital Medical University, Beijing 100000, China; ^3^Department of Neurology, The First Affiliated Hospital, College of Clinical Medicine of Henan University of Science and Technology, Luoyang 471003, China

## Abstract

Mitophagy and oxidative stress play important roles in Parkinson's disease (PD). Dysregulated mitophagy exacerbates mitochondrial oxidative damage; however, the regulatory mechanism of mitophagy is unclear. Here, we provide a potential mechanistic link between c-Abl, a nonreceptor tyrosine kinase, and mitophagy in PD progression. We found that c-Abl activation reduces the interaction of prohibitin 2 (PHB2) and microtubule-associated protein 1 light chain 3 (LC3) and decreases the expressive level of antioxidative stress proteins, including nuclear factor erythroid 2-related factor 2 (Nrf2), NADPH quinone oxidoreductase-1 (NQO-1), and the antioxidant enzyme heme oxygenase-1 (HO-1) in 1-methyl-4-phenylpyridinium- (MPP^+^-) lesioned SH-SY5Y cells. Importantly, we found that MPP^+^ can increase the expression of phosphorylated proteins at the tyrosine site of PHB2 and the interaction of c-Abl with PHB2. We showed for the first time that PHB2 by changing tyrosine (Y) to aspartate (D) at site 121 resulted in impaired binding of PHB2 and LC3 in vitro. Moreover, silencing of PHB2 can decrease the interaction of PHB2 and LC3 and exacerbate the loss of dopaminergic neurons. We also found that STI 571, a c-Abl family kinase inhibitor, can decrease dopaminergic neuron damage and ameliorate MPTP-induced behavioral deficits in PD mice. Taken together, our findings highlight a novel molecular mechanism for aberrant PHB2 phosphorylation as an inhibitor of c-Abl activity and suggest that c-Abl and PHB2 are potential therapeutic targets for the treatment of individuals with PD. However, these results need to be further validated in PHB2 Y121D mice.

## 1. Introduction

Parkinson's disease is the second most common neurodegenerative disease and is characterized by progressive degeneration of dopaminergic neurons, leading to severe motor complications [1]. The high metabolic activity, low antioxidant capacity, and nonreplication properties of dopaminergic neurons render them more susceptible to oxidation and reactive oxygen species (ROS) during oxidative phosphorylation, leading to oxidative stress damage [2]. MPP^+^ is the most widely used inducer of PD like pathogenicity in in vitro studies [3] and can be formed by the oxidation of MPTP by monoamine oxidase B in vivo. MPP^+^ can directly inhibit the activity of mitochondrial respiratory enzyme complex I, reduce mitochondrial membrane potential, stimulate oxidative stress, increase reactive oxygen species (ROS) levels, destroy the mitochondrial membrane structure, and cause neurodegeneration [4].

Oxidative stress is closely related to mitophagy. Mitophagy selectively removes damaged mitochondria [5, 6] and reduces oxidative stress damage, thereby protecting dopaminergic neurons [7, 8]. In the autopsy of patients with Parkinson's disease, disordered mitochondrial autophagy clearance in the brain was found [9]. At present, research on mitophagy-related proteins in Parkinson's disease mainly focuses on the outer mitochondrial membrane, and few reports on inner mitochondrial membrane proteins are available. The newly discovered mitochondrial inner membrane protein prohibitin 2 (PHB2) has been confirmed to be involved in mitophagy [10, 11], but no report indicates that PHB2 is involved in mitophagy in Parkinson's disease.

PHB2 is a newly discovered mitochondrial inner membrane protein related to aging [12, 13], proliferation [14], degeneration [15, 16], and metabolic diseases [17, 18]. It is reported that PHB2-induced mitophagy suppresses tubular epithelial cell damage and NLRP3 inflammatory body activation by regulating mitochondrial dysfunction [19]. Another study also suggests that PHB2 is required for cholestasis-induced mitophagy via LC3 onto the injured mitochondria [20]. Autophagy is highly selective, and the heart of this selectivity lies in the LC3 interaction region (LIR) motif, which ensures the targeting of autophagy receptors to LC3 anchored on autophagic vacuole membranes [21]. When oxidative stress damage leads to the rupture of the outer mitochondrial membrane, PHB2 localized in the mitochondrial inner membrane can bind to LC3 and promote autophagy of damaged mitochondria. The key structural domain of the PHB2 and LC3 interaction (LIR) is the 121-124 position (YQRL) of PHB2. However, the PHB2 LIR domain Y121A/L124A mutation inhibits PHB2 binding to LC3 and causes impaired mitophagy [20, 22]. However, whether PHB2 and its gene mutations are involved in the pathological process of PD has not been reported.

c-Abl is a member of the Src family of nonreceptor tyrosine kinases that exists in the cytoplasm, nucleus, and mitochondrial matrix. It targets mitochondria to regulate mitochondrial dysfunction and cell death [23]. The protein expression of c-Abl in most cells is an important part of intracellular protein interactions and phosphorylation [24]. Under physiological conditions, c-Abl exists in a self-inhibiting state. Under complex molecular interactions, c-Abl can be activated by autophosphorylation in Y421 and Y245 [25, 26]. A series of studies have shown that c-Abl activation is closely related to PD [27, 28]. c-Abl can also phosphorylate Parkin and inhibit its E3 ligase activity, leading to the accumulation of neurotoxins [29]. STI 571 can reduce dopaminergic neuron loss and ameliorate motor defects in PD mouse models by inhibiting c-Abl phosphorylation [30].

Therefore, we mainly aimed to identify whether c-Abl regulated the binding of PHB2 and LC3 to affect mitophagy and the role of the c-Abl-PHB2 pathway in the regulation of dopaminergic neuron death. In this study, an MPP^+^-exposed SH-SY5Y cell and an MPTP-induced acute Parkinson's disease mouse model were used. We determined that PHB2 can be phosphorylated by tyrosine kinase and interact with c-Abl by immunoprecipitation assay. In addition, we simulated the permanent phosphorylation model of PHB2 by changing tyrosine (Y) to aspartate (D) at site 121 and evaluated the effect of PHB2 Y121D mutation on the binding of PHB2 to LC3. Moreover, we explored the role of PHB2-mediated mitophagy in the pathogenesis of PD in C57 mice injected with PHB2-shRNA. Taken together, the novel c-Abl-PHB2-autophagy axis may be a potential and prospective therapeutic target for PD.

## 2. Materials and Methods

### 2.1. Cell Culture and Treatment

SH-SY5Y cells, which were obtained from Sun Ye San University (Guangzhou, China), were grown in DMEM/F12 (Corning, New York, USA) with 10% fetal bovine serum (Gibco, Gaithersburg, USA) and 1% penicillin-streptomycin. SH-SY5Y cells were maintained at 37°C in an incubator containing 5% carbon dioxide. To produce oxidative stress, 100 mM MPP^+^ (Sigma-Aldrich, St. Louis, USA) was freshly prepared in deionized water as a stock solution before each experiment. SH-SY5Y cells were incubated with specific concentrations of MPP^+^ (1 mM) for 24 h. The c-Abl inhibitor STI-571 (Gleevec, imatinib mesylate; Novartis Pharma AG) was added to the cells at 10 *μ*M for 6 h before toxin treatment.

### 2.2. Animals

Six- to eight-week-old C57BL/6J male mice (purchased from the Beijing Vital River Laboratory Animal Technology Co., Ltd., Beijing, China) were maintained under conditions of a 12 h light/dark cycle at 23°C and were provided with food and water ad libitum in the Animal Care Facility at the Institute of Biophysics (Beijing, China). All experiments involving animals were approved by and conformed to the guidelines of the institutional animal care and use committee at the Institute of Biophysics of the Chinese Academy of Sciences (Beijing, China).

### 2.3. Drug Treatment In Vivo

**Figure 1:**
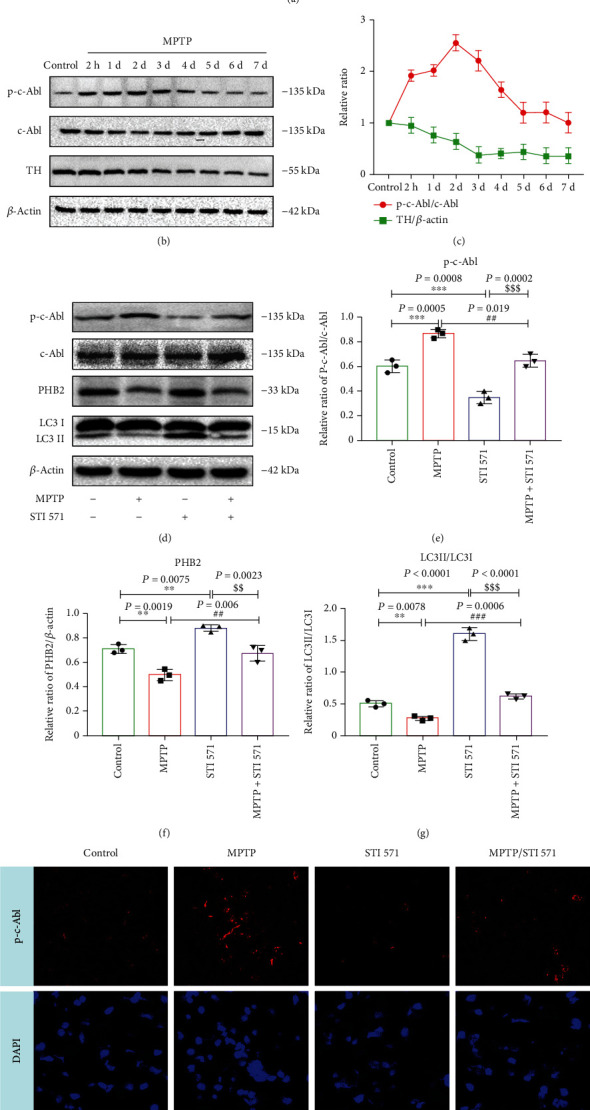
STI 571 increases PHB2-mediated mitophagy in PD mice. (a) Flow chart of the animal experiment. (b) C57BL-6J mice were treated with saline or MPTP (20 mg/kg, iv, every 2 hours). Brain tissue was taken at 2 h and 7 d after MPTP injection. p-c-Abl Y245 and TH protein expressions by western blot. (c) The normalized levels of p-c-Abl Y245 and TH. (d) C57BL-6J mice were treated with STI 571 (30 mg/kg intraperitoneal injection) and MPTP, and brain tissue was taken at 2 day; c-Abl, p-c-Abl, PHB2, and LC3 protein expressions change. (e–g) p-c-Abl Y245, PHB2, and LC3II/LC3I protein concentrations change by quantitative analysis. (h) C57BL-6J mice were treated with STI 571 and MPTP, and brain tissue was taken at 2 day. Fluorescence immunohistochemical analysis of p-c-Abl Y245 in the substantia nigra pars compacta of the middle brain. Red fluorescence: p-c-Abl Y245; blue fluorescence: DAPI. Scale bar = 20 *μ*m (^∗∗∗^*P* < 0.001 and ^∗∗^*P* < 0.01 compared with the control group; ^###^*P* < 0.001 and ^##^*P* < 0.01 compared with the MPTP group; ^$$$^*P* < 0.001 and ^$$^*P* < 0.01 compared with the STI 571 group; *n* = 3, mean ± SEM).

The mice were randomly assigned to four groups (*n* = 5/group): (1) control, (2) MPTP, (3) STI 571, and (4) MPTP+STI 571. The first group, i.e., control (*n* = 5), was treated with normal saline (i.p.). MPTP (20 mg/kg) was prepared by dissolving it in 0.9% saline. The mice were injected (i.p.) with MPTP (20 mg/kg) every 2 h for a total of four doses over an 8 h period in 1 d of the interval to induce PD in the second group [3] (*n* = 5). The third group (*n* = 5) was given four injections (i.p.) of STI 571 (30 mg/kg) every other day. The fourth group (*n* = 5) was given four injections (i.p.) of STI 571 (30 mg/kg) every other day and was given MPTP injections between the second and third injections of STI 571. Seven days after the last injection of MPTP, the rotarod test and water maze test were used to evaluate behavior. The mice were sacrificed after all behavioral tests (Figure 1(a)). Brain tissue was collected and analyzed by western blotting. Animals were perfused with 4% paraformaldehyde in 0.1 mM phosphate buffer (pH 7.4), and 5 *μ*M coronal paraffin sections were prepared for immunohistochemical analysis [31].

### 2.4. Establishment of a Stable PHB2-shRNA Cell Line

Lentiviral particles expressing PHB2-shRNA or control-shRNA were obtained from Santa Cruz (Dallas, USA). Following the manufacturer's protocol, SH-SY5Y cells were placed in a 12-well plate 24 hours before viral infection. When the cells were 50% confluent, they were infected by adding 5 × 10^4^ U PHB2-shRNA or control-shRNA lentivirus particles into the culture containing 5 *μ*g/mL polybrene (Santa Cruz, Dallas, USA) to infect cells. Stable clones were selected for 1 week by using 5 *μ*g/mL puromycin.

### 2.5. Animal Surgery

The shRNA-PHB2 mouse model was prepared by microinjection of lentivirus shRNA-PHB2, at the titers 1 × 10^8^ TU/mL (GeneChem, Shanghai, China). The mice were randomly assigned to three groups (*n* = 5/group): (1) sham, (2) control-shRNA, and (3) PHB2-shRNA. All mice were anesthetized by an intraperitoneal (i.p.) injection of pentobarbital (50 mg/kg) and permitted to breathe spontaneously during surgery. A hole was drilled in the appropriate location for the striatum in the sham group without injection. The PHB2-shRNA group and control-shRNA group followed these steps. Briefly, animals were anesthetized and placed in a stereotactic head frame. After making a midline incision in the scalp, a hole was drilled in the appropriate location for the striatum at the right side of the skull. Two microliters of the viral vector was injected at a rate of 0.2 *μ*L/min with a 33-gauge needle on a 10 *μ*L Hamilton syringe at the following coordinates: anteroposterior –0.8, mediolateral +2.0, and dorsal-ventral –3.6 relative to bregma. Mice were then sacrificed 8 weeks after the injections for immunohistochemical or biochemical analysis.

### 2.6. Detection of Intracellular Reactive Oxygen Species

The assay was performed to analyze the levels of ROS in SH-SY5Y by using fluorescent dye 2′,7′-dichlorofluoresce diacetate (DCFH-DA, Beyotime Biotechnology, Shanghai, China). The collected cells were suspended in DCFH-DA (10 *μ*mol/L) diluted in a serum-free medium. Cells were incubated at 37°C for 20 minutes and then washed. Fluorescence was measured using a personal flow cytometry analyzer (Accuri C6, BD, USA).

### 2.7. Transmission Electron Microscope Observation of Mitochondria

More than 10^6^ SH-SY5Y cells were quickly scraped off with a cell scraper and centrifuged at 1500–3000 for 5–10 min, and 2–3 samples (approximately the size of rice grains) visible to the naked eye at the bottom of the tube were collected. The samples were treated with glutaraldehyde electron microscope fixative, embedded in resin, ultrathin sections, stained with lead citrate and uranium diacetate, and observed under an electron microscope.

### 2.8. Western Blotting Analysis

The total proteins of cells or tissues were collected using RIPA lysis buffer (Beyotime, Beijing, China). The protein of equal quality was electrophoresed on an SDS-PAGE gel and transferred to PVDF membranes (PVDF, Millipore Immobilon-P^SQ^). The PVDF membranes were incubated overnight at 4°C in solutions with primary antibodies (1 : 2000) against PHB2, LC3 (Abcam, Cambridge, UK), c-Abl (Santa Cruz, Dallas, USA), and p-Y245 c-Abl (Cell Signaling Technology, Danvers, USA); primary antibodies (1 : 2000) against Nrf2, HO-1, and NQO-1 (Cell Signaling Technology, Danvers, USA); and primary antibodies (1 : 5000) against *β*-actin (CWBio, Beijing, China), washed with PBST three times and incubated with a 1 : 5000 dilution of the secondary antibody (CWBio, Beijing, China) at room temperature for 1 h. Negative controls were prepared by excluding the primary antibodies. Images of the labeled membranes were analyzed using the ImageJ software (National Institutes of Health, USA).

### 2.9. Coimmunoprecipitation

Coimmunoprecipitation kit (Thermo Scientific, MA, USA) was used. For coimmunoprecipitation, cells were lysed in IP buffer (pH 7.4, 0.025 M Tris, 0.15 M NaCl, 0.001 M EDTA, 1% NP-40, 5% glycerol) for 20-30 min and then centrifuged at 13000 rpm for 10 min. Lysates were precleared with coupling resin and incubated with specific antibody binding for 2–4 hours at 4°C. The immunoprecipitate was finally eluted using an elution buffer containing a primary amine and analyzed by immunoblotting.

### 2.10. Rotarod Test

Two people handled the mice, neither of whom knew the task of the treatment group. The ability of the mouse to balance movement was measured by a 5 cm diameter screw rod with five runways. The mouse was placed on the rotameter in advance for several rounds of training to adapt to the rotameter program. Rotary experiments were performed on mice for 4 consecutive days from the seventh day after MPTP injection. The mice were placed on the horizontal bar at a starting speed of 0 rpm and an acceleration of 6 rpm/min, and the time from the rotation axis to the falling of the mice was recorded. The mice remaining on the device after 600 s were removed, and the time was recorded as 600 s. Each mouse was tested three times a day (1-hour intervals), and the average value was taken for statistical analysis.

### 2.11. Morris Water Maze Test

The water maze device is a black circular tank with a diameter of 120 cm, a height of 50 cm, and a water depth of 30 cm. Through the recording system, a black platform with a diameter of 10 cm was placed in the water maze (SMART Panlab, Harvard Bioscience, Shanghai, China). The top of the platform is approximately 2 cm below the water surface. The tank is divided into four equal parts (A-D regions) and a platform region (T region). The platform is located in area A. In the first phase (training phase, day 9), each mouse was gently placed in a water maze in area C facing the wall for 90 seconds. If the platform was not found during the training, the mice were guided to the platform and maintained for 20 s for observation and learning. In the second stage (days 10–15), each mouse was gently placed in the water maze of zone C facing the water tank wall to seek a platform. If the rats successfully found the platform and remained in the T area for 2 s, the time was recorded. If the platform was not found within 90 s, the recording time was 90 s, and then, the mice were guided to the platform and maintained for 20 s for observation and learning. In the third phase (platform crossing phase, day 16), the platform was removed, and each mouse was gently placed in the water maze of area C. The number of mice crossing the T area within 90 seconds was recorded. The learning ability and spatial memory ability of the mice were evaluated according to the platform search latency and cross-platform times [32].

### 2.12. Immunofluorescence

SH-SY5Y cells were grown on glass coverslips and treated as described previously. Then, cells or brain tissue sections were fixed with 4% paraformaldehyde, permeabilized with 0.25% Triton X-100, and blocked with goat serum for 30 minutes. After incubation with primary antibodies (1 : 1000) against PHB2, LC3, p-Y245 c-Abl, *α*-syn, and TH at 4°C overnight, the cells and sections were washed three times with PBS, incubated with DAPI and fluorescent secondary antibody in blocking buffer for 1 h at 37°C. After washing with PBS three times, all images were observed by an LSM 780 confocal microscope (Zeiss, Oberkochen, Germany). TH positive neuron counting method refers to a previously published article [33].

### 2.13. Statistics and Analysis

The graphs and data from the experiments were drafted and analyzed with the statistical program GraphPad Prism 7. Comparisons between groups were evaluated by one-way analysis of variance with Tukey's multiple comparison test. Two-way analyses of variance and Bonferroni's post hoc tests were used to analyze differences between treatment groups. Data are presented as mean ± SEM, and a difference of *P* < 0.05 was considered statistically significant.

## 3. Results

### 3.1. MPP^+^ Induced Phosphorylation of c-Abl and Decreased Oxidative Stress-Related Protein Expression in SH-SY5Y Cells

**Figure 2:**
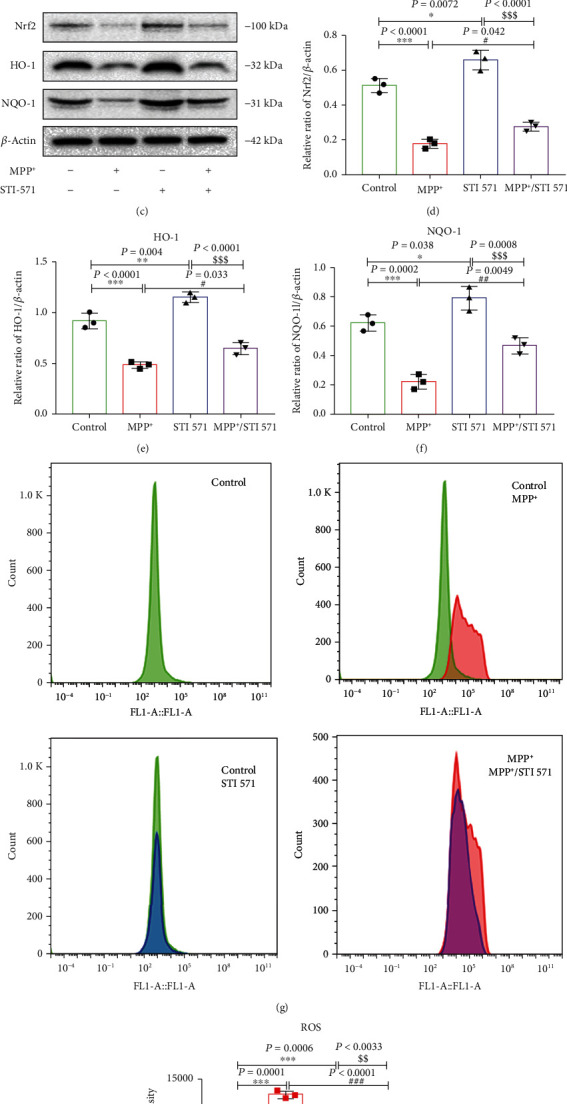
MPP^+^ induces c-Abl phosphorylation and increases oxidative stress. (a) c-Abl and p-c-Abl protein expressions change with 1 mM MPP^+^ and pretreatment with 1 *μ*M STI 571 for 6 hours. (b) p-c-Abl protein concentration changes by quantitative analysis. (c) NQO-1, Nrf2, and HO-1 protein expressions change with 1 mM MPP^+^ and/or pretreatment with 1 *μ*M STI 571 for 6 hours. (d–f) NQO-1, Nrf2, and HO-1 protein concentrations change by quantitative analysis. (g, h) Effects of MPP^+^ and STI 571 on intracellular ROS in SH-SY5Y cells (^∗∗∗^*P* < 0.001, ^∗∗^*P* < 0.01, and ^∗^*P* < 0.05 compared with the control group; ^###^*P* < 0.001, ^##^*P* < 0.01, and ^#^*P* < 0.05 compared with the MPP^+^ group; ^$$$^*P* < 0.001 and ^$$^*P* < 0.01 compared with the STI 571 group; *n* = 3, mean ± SEM).

The cell model established by MPP^+^-exposed SH-SY5Y cells has become a classic cell model of PD in vitro. The results showed that the expression of phosphorylated c-Abl (p-c-Abl Y245) was increased in MPP^+^ exposed cells when compared to the control group, and pretreatment with STI 571 reversed this increase (Figures 2(a) and 2(b)). Next, we investigated the effect of c-Abl phosphorylation on MPP^+^-induced oxidative stress. MPP^+^ significantly decreased the protein expression of HO-1, NQO-1, and Nrf2, and STI 571 pretreatment increased the expression of antioxidative stress proteins (Figures 2(c)–2(f)). Moreover, the ROS levels were increased in MPP^+^ exposed cells, and STI 571 treatment can inhibit the increase (Figures 2(g) and 2(h)). Together, these data suggested that MPP^+^ can increase the level of c-Abl phosphorylation and aggravate oxidative stress injury and that inhibition of c-Abl phosphorylation by STI 571 can attenuate oxidative stress injury.

### 3.2. The c-Abl Interaction with PHB2 Decreased the Mitophagy in the MPP^+^-Induced PD Cell Model

**Figure 3:**
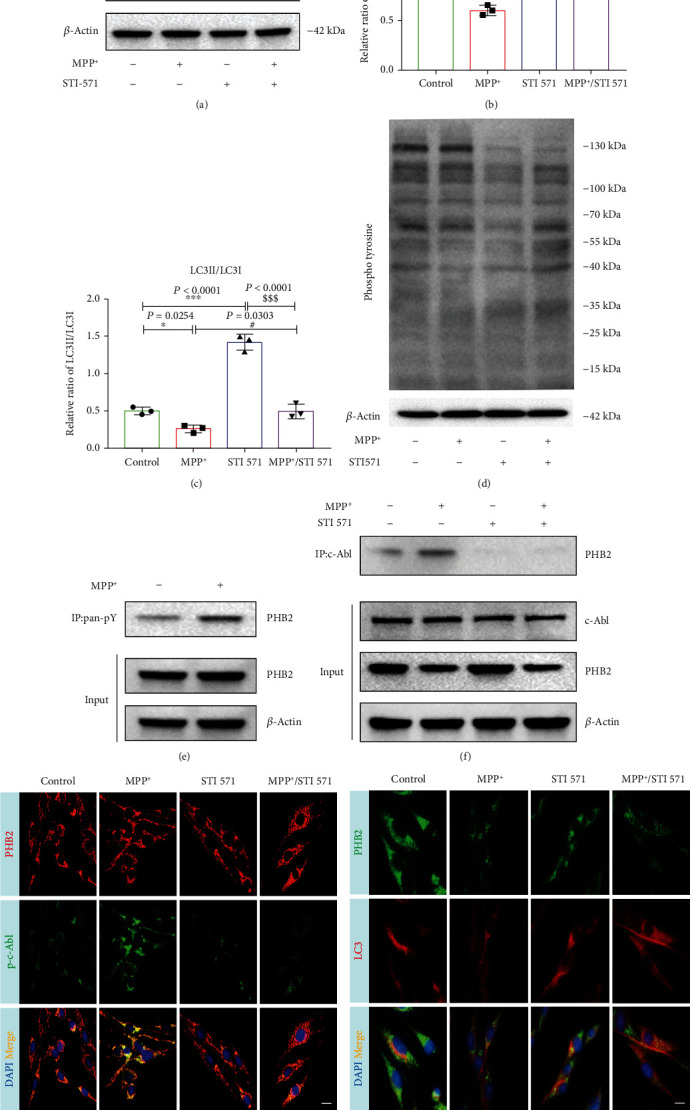
PHB2 as a major substrate of c-Abl in PD cell model. (a) LC3II/LC3I and PHB2 protein expressions change with 1 mM MPP^+^ and pretreatment with 1 *μ*M STI 571 for 6 hours. (b, c) LC3II/LC3I and PHB2 protein concentrations change by quantitative analysis. (d) Phosphor-tyrosine protein expression changes with 1 mM MPP^+^ and pretreatment with 1 *μ*M STI 571 for 6 hours. (e) The cells were treated with MPP^+^; then, the cell lysates were immunoprecipitated using an antiphosphotyrosine antibody followed by immunoblotting using an antibody against PHB2. (f) Cells were treated with MPP^+^ and STI 571, followed by immunoprecipitation of cell lysates with c-Abl antibody, followed by immunoblotting with antibody against PHB2. (g) SH-SY5Y cells were treated with MPP^+^ and STI 571; p-c-Abl and PHB2 protein immunofluorescence changes. Red fluorescence: PHB2; green fluorescence: p-c-Abl; blue fluorescence: DAPI. (h) SH-SY5Y cells were treated with STI 571 and MPP^+^; PHB2 and LC3 protein immunofluorescence changes. Red fluorescence: LC3; green fluorescence: PHB2; blue fluorescence: DAPI. Scale bar = 20 *μ*m (^∗∗∗^*P* < 0.001 and ^∗^*P* < 0.05 compared with the control group; ^###^*P* < 0.001 and ^#^*P* < 0.05 compared with the MPP^+^ group; ^$$$^*P* < 0.001 and ^$^*P* < 0.05 compared with the STI 571 group; *n* = 3, mean ± SEM).

Next, we investigated the mechanisms by which c-Abl modulates the mitophagy of PHB2. MPP^+^ increased the protein expression of p-c-Abl Y245, accompanied by a decrease in the LC3II/LC3I ratio, while STI 571 decreased the protein expression of p-c-Abl Y245 and increased the LC3II/LC3I ratio (Figures 3(a) and 3(c)). Interestingly, we found that STI 571 can increase the protein expression of PHB2 (Figures 3(a) and 3(b)). To determine whether STI 571 is involved in protein tyrosine phosphorylation, we used a total tyrosine phosphorylation antibody to study the effects of STI 571 in MPP^+^-induced SH-SY5Y cells. The results showed that STI 571 could significantly inhibit the expression of tyrosine phosphorylation (Figure 3(d)). To determine whether PHB2 can be phosphorylated by tyrosine kinases, we harvested cells 24 hours after MPP^+^ treatment and coimmunoprecipitated cell lysates with agarose beads. Immunoprecipitates were detected by immunoblotting with anti-PHB2 antibody. The results showed that MPP^+^ treatment increased the tyrosine phosphorylation of PHB2 (Figure 3(e)). To determine whether c-Abl interacts with PHB2, cell lysates were used for coimmunoprecipitation after MPP^+^ and STI 571 treatment. The results showed that MPP^+^ treatment increased the interaction of c-Abl with PHB2, while STI 571 decreased the interaction of c-Abl with PHB2 (Figure 3(f)). Immunofluorescence staining showed that the colocalization of p-c-Abl Y245 and PHB2 was significantly increased by MPP^+^ treatment. When STI 571 inhibited the activity of c-Abl, the colocalization of p-c-Abl Y245 and PHB2 was reduced (Figure 3(g)). When MPP^+^ phosphorylated c-Abl, the colocalization of PHB2 and LC3 was significantly decreased. However, colocalization was increased with STI 571 (Figure 3(h)). Our results suggested that PHB2 was a c-Abl substrate and that MPP^+^-induced c-Abl phosphorylation may affect mitophagy by altering the tyrosine phosphorylation of PHB2, and STI 571 can increase the protein expression of PHB2 and increase mitophagy.

### 3.3. Mutation of PHB2 Y121D Reduced the Binding Efficiency with LC3

**Figure 4:**
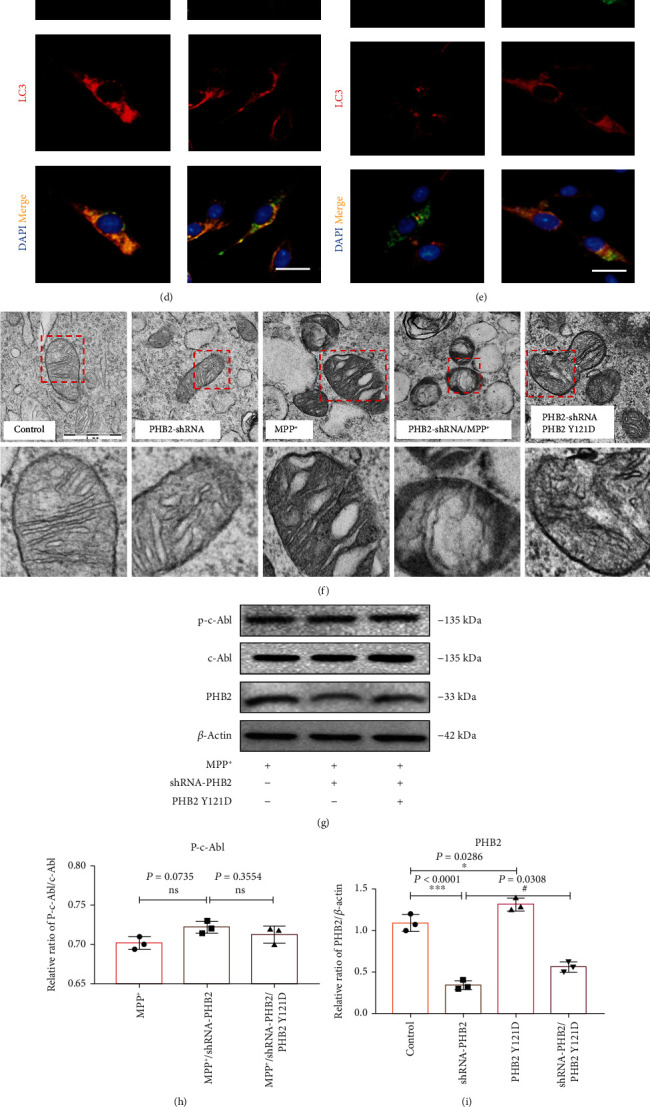
Mutation of PHB2 Y121D reduces binding efficiency to LC3. (a) PHB2 Y121D transiently transfects normal or low PHB2-expressing SH-SY5Y cell lines; p-c-Abl and PHB2 protein expressions change. (b, c) P-c-Abl and PHB2 protein concentrations change by quantitative analysis. (d, e) The SH-SY5Y cell line with low expression stably screened by shRNA-PHB2 or normal SH-SY5Y was transiently transfected with PHB2Y121D with a GFP fluorescent label; PHB2 used Proteintech 66424-1-Ig (mouse monoclonal) green fluorescence with Alexa Fluor 488 and PHB2 Y121 D with a GFP fluorescence label, and LC3 used Abcam ab192890 (rabbit monoclonal) red fluorescence with Alexa Fluor 555, DAPI staining of the nucleus. Scale bar = 20 *μ*m. (f) Effects of low expression of PHB2 and PHB2 Y121 mutation on mitochondria in SH-SY5Y cells. The red box shows the magnified picture of mitochondria. Scale bar = 1 *μ*m. (g) PHB2 Y121D transiently transfects normal or low PHB2-expressing SH-SY5Y cell lines, and then, the cells were treated with MPP^+^; p-c-Abl and PHB2 protein expressions change. (h, i) P-c-Abl and PHB2 protein concentrations change by quantitative analysis. (j) The SH-SY5Y cell line induced by MPP^+^ with low expression stably screened by shRNA-PHB2 or normal SH-SY5Y was transiently transfected with PHB2Y121D with a GFP fluorescent label; PHB2 used Proteintech 66424-1-Ig (mouse monoclonal) green fluorescence with Alexa Fluor 488 and PHB2 Y121 D with a GFP fluorescence label, and LC3 used Abcam ab192890 (rabbit monoclonal) red fluorescence with Alexa Fluor 555, DAPI staining of the nucleus. Scale bar = 20 *μ*m (^∗∗∗^*P* < 0.001 and ^∗^*P* < 0.05 compared with the control group; ^#^*P* < 0.05 compared with the shRNA-PHB2 group or shRNA-PHB2 with MPP^+^ group; ns: not significant in the control group; *ns*: not significant in the shRNA-PHB2 group; *n* = 3, mean ± SEM).

To determine whether phosphorylation of the PHB2 Y121 site is involved in mitophagy, SH-SY5Y cell lines stably transfected with shRNA-PHB2 and PHB2 Y121D were transiently transfected with GFP fluorescence labels. The results showed that overexpression of PHB2 Y121D increased the protein expression of PHB2 but had no significant effect on the protein expression of p-c-Abl Y245 (Figures 4(a)–4(c) and Figures S1(a)-S1(c)). Overexpression of PHB2 Y121D (GFP green fluorescence) significantly decreased the colocalization of LC3 and PHB2, which was consistent with the results of shRNA-PHB2 (Figures 4(d) and 4(e)). Moreover, we further observed the mitochondrial damage in shRNA-PHB2-treated cells by electron microscopy and found that PHB2 silence could cause slight damage to the mitochondrial inner ridge structure. MPP^+^ caused mitochondrial shrinkage. PHB2 silence under MPP^+^ treatment caused swelling of the mitochondrial inner ridge. Interestingly, we transformed the PHB2 Y121D plasmid into a low-expressing PHB2 cell line and found that mitochondrial damage was not improved by increasing PHB2 expression (Figure 4(f)). The overexpression of PHB2 Y121D increased the protein expression of PHB2 but had no significant effect on the protein expression of p-c-Abl Y245 in the PD cell model (Figures 4(g)–4(i)). The same result showed that overexpression of PHB2 Y121D (GFP green fluorescence) significantly decreased the colocalization of LC3 and PHB2, which was consistent with the results of shRNA-PHB2 in the PD cell model (Figure 4(j)). Together, these data suggested that PHB2 phosphorylation reduced mitophagy.

### 3.4. Silencing of PHB2 Resulted in Decreased Mitophagy and Increased Loss of Dopaminergic Neurons In Vivo

**Figure 5:**
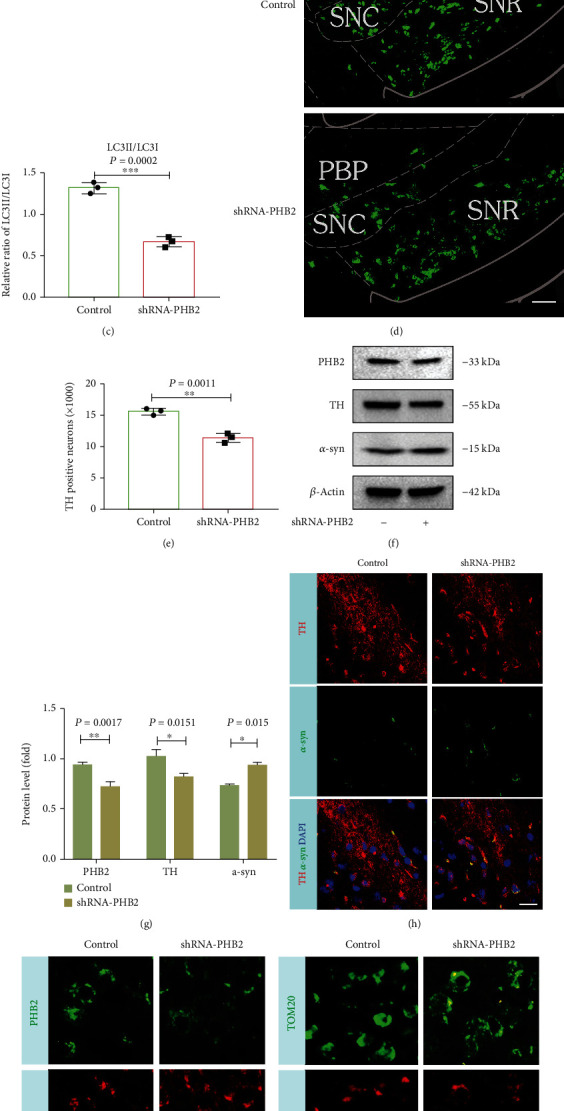
Silencing of PHB2 inhibits mitophagy and increased neuronal loss in vivo. (a) PHB2 and LC3II/LC3I protein expressions change in C57BL-6J mice brain tissue at 8 weeks after PHB2-shRNA lentivirus injection. (b, c) LC3II/LCI and PHB2 protein concentrations change by quantitative analysis. (d, e) After shRNA-PHB2 lentivirus injection at 8 weeks, TH immunofluorescence (green fluorescence) in the substantia nigra pars compacta of the middle brain was analyzed statistically with the number of TH-positive neurons. (f, g) PHB2, TH, and *α*-syn protein expressions change and quantitative analysis in C57BL-6J mouse brain tissue at 8 weeks after PHB2-shRNA lentivirus injection. (h) Fluorescence immunohistochemical colocalization analysis of TH and in the substantia nigra pars compacta of the middle brain. Green fluorescence indicates *α*-syn, red fluorescence indicates TH, and blue fluorescence indicates DAPI. (i, j) Fluorescence immunohistochemical colocalization analysis of LC3, PHB2, and TOM20 in the substantia nigra pars compacta of the middle brain. Green fluorescence indicates PHB2 and TOM20, red fluorescence indicates LC3, and blue fluorescence indicates DAPI. Scale bar = 20 *μ*m (^∗∗∗^*P* < 0.01, ^∗∗^*P* < 0.01, and ^∗^*P* < 0.05 compared with the control group; *n* = 3, mean ± SEM).

To demonstrate the role of PHB2 in vivo, shRNA-PHB2 lentivirus was injected into the striatum of C57BL-6J mice, and western blotting and immunohistochemistry were performed eight weeks after injection. We found that silencing of PHB2 decreased the LC3II/LC3I ratio (Figures 5(a)–5(c)) and increased damage to dopaminergic neurons (Figures 5(d) and 5(e)). However, the expression of PHB2 and LC3 in the sham and control-shRNA groups was not statistically significant (Figures S2(a)-S2(c)), and there was no significant difference in the number of TH-positive neurons (Figures S2(d) and S2(e)). Further study found that inhibition of PHB2 decreased the protein expression of TH and increased the protein expression of *α*-syn (Figures 5(f) and 5(g)). TH and *α*-syn fluorescent double staining showed that shRNA-PHB2 striatal injection could increase the expression of *α*-syn (Figure 5(h)). Brain-targeted injection of shRNA-PHB2 decreased TOM20 and PHB2 with LC3 colocalization (Figures 5(i) and 5(j)). These data suggested that PHB2 phosphorylation reduced mitophagy. These data suggested that PHB2 was involved in mitophagy and affected PD pathology.

### 3.5. Inhibition of c-Abl Phosphorylation Increased PHB2-Mediated Mitophagy in MPTP-Induced PD Mice

Next, we investigated whether c-Abl phosphorylation activates PHB2 in PD mice. MPTP (20 mg/kg i.p. 4 times a day, 2 hours apart) and/or STI 571 (30 mg/kg i.p.) was used in mice (Figure 1(a)). Brain tissues were dissected daily for western blot analysis from 2 h to 7 d after MPTP treatment. The results showed that the ratio of p-c-Abl/c-Abl increased from 2 hours to 2 days after MPTP treatment (Figures 1(b) and 1(c)); the protein expression of tyrosine hydroxylase (TH) decreased after MPTP treatment for 2 hours, and it remained low for 5-7 days (Figures 1(b) and 1(c)). To investigate the effect of c-Abl activation on PHB2 and mitophagy, we selected mice for experiments after MPTP treatment for 2 days. Western blot analysis showed that STI 571 decreased the protein expression of p-c-Abl Y245 and increased the LC3II/LC3I ratio, and STI 571 increased the expression of PHB2 (Figures 1(d)–1(g)). Immunofluorescence staining also showed that STI 571 could reduce c-Abl phosphorylation (Figure 1(h)). Taken together, these data suggested that c-Abl affected PHB2-mediated mitophagy in PD.

### 3.6. Inhibition of c-Abl Alleviated MPTP-Induced Dopaminergic Neuron Loss and Motor Defects

**Figure 6:**
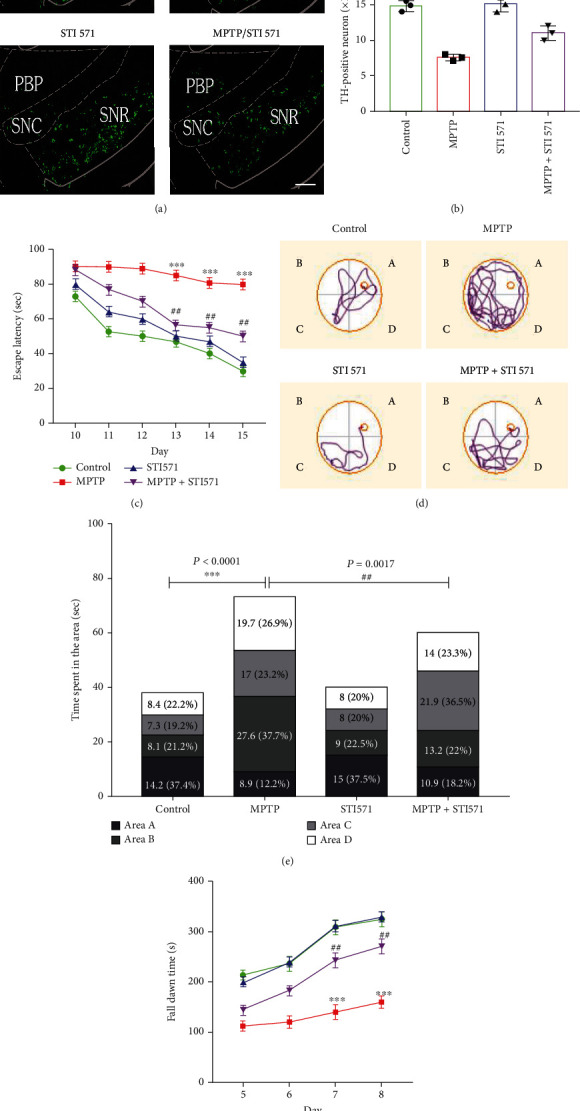
STI 571 alleviates MPTP-induced dopaminergic neuron loss and motor defects. (a, b) All behavioral experiments were completed, and TH immunofluorescence (green fluorescence) in the substantia nigra pars compacta of the middle brain was analyzed statistically to determine the number of TH-positive neurons. (c) The learning ability of C57BL-6J mice was detected by an escape latency test from 10 d to 15 d. (d) Trajectory map of the C57BL-6J mouse search platform (the platform is in area A, and mice enter the pool from area C). (e) The platform was removed at 16 days. Detection of spatial memory using the water maze test. The results were expressed as the latency of time between entering the water and finding the platform. (f) Detection of muscle capacity using the rotarod test. The results are expressed as the time taken by mice to fall from the rotarod instrument (^∗∗∗^*P* < 0.001, compared with the control group; ^##^*P* < 0.01 compared with the MPTP group; ^$$^*P* < 0.01 compared with the STI 571 group; *n* = 3, mean ± SEM).

MPTP can cause mitochondrial oxidative stress damage in substantia nigra dopaminergic neurons, leading to impairment of movement, learning, and memory. Dopaminergic neuron loss was more pronounced after MPTP treatment, whereas STI 571 ameliorated dopaminergic neuron loss (Figures 6(a) and 6(b)). In the rotarod experiment, we performed base line rotarod test (Table S1 and Figure S3). MPTP treatment significantly shortened the duration of the mice on the roller, whereas STI 571 prolonged the time on the roller in MPTP-treated mice (Figure 6(f)). The escape latency of MPTP-treated mice did not show a clear downward trend, and the escape latency of the STI 571-treated group decreased significantly on days 2 and 3 of the water maze test and reached a lower level on day 6 (Figures 6(c) and 6(d)). Six days after the water maze experiment, the platform was removed, and the percentage of time spent in area A (where the platform was) was significantly reduced in the MPTP group, and STI 571 attenuated MPTP-induced learning and spatial memory impairments (Figure 6(e)). Overall, inhibition of c-Abl phosphorylation reduced MPTP-induced loss of dopaminergic neurons and improved motor, learning, and spatial memory in PD mice.

## 4. Discussion

In our study, MPP^+^ significantly increased the c-Abl phosphorylation level of SH-SY5Y cells and aggravated oxidative stress injury. We also found that the c-Abl inhibitor STI 571 can upregulate the protein expression of Nrf2, HO-1, and NQO-1 and play a role in antioxidative stress. Upregulation of the LC3II/LC3I ratio suggested that STI 571 enhanced autophagy by inhibiting c-Abl phosphorylation. By coimmunoprecipitation, we demonstrated that MPP^+^ could increase the expression of phosphorylated protein at the tyrosine site of PHB2 and increase the interaction of c-Abl with PHB2. Moreover, STI 571 could reduce these phenomena. In the PHB2 phosphorylation model PHB2 Y121D, we found that the colocalization of PHB2 and LC3 was reduced, which was consistent with the results of shRNA-PHB2. In addition, we also confirmed that silencing of PHB2 reduced the loss of dopaminergic neurons in the shRNA-PHB2 lentivirus striatum injection mouse model. Finally, we confirmed that c-Abl was also activated in MPTP-induced PD mice, and STI 571 alleviated MPTP-induced dopaminergic neuron loss and motor defects. This work significantly advances our understanding of how mitophagy and oxidative stress are regulated by the c-Abl-PHB2 pathway.

Recent studies have found that c-Abl can affect the occurrence and progression of Parkinson's disease. The evidence indicated that c-Abl phosphorylated Parkin at Tyr^143^, and STI 571 maintained Parkin in a catalytically active and neuroprotective state by preventing the tyrosine phosphorylation of Parkin [30]. It was also found that STI 571 rescued the autophagy-lysosomal pathway (ALP) function through facilitating the nuclear translocation of transcription factor EB (TFEB) and protected against MPP^+^-induced neuronal cell death [34]. In an MPTP-induced acute PD model, treatment of mice with STI 571 reduced the loss of dopaminergic neurons and ameliorated the locomotive defects by preventing p38*α* phosphorylation [31]. A dual-blind clinical trial of the c-Abl inhibitor nilotinib failed to show its clinical efficacy in patients with PD [35, 36], which is likely a result of incomplete inhibition of c-Abl activation [37]. The mounting evidence indicates that c-Abl has the potential to be a disease-modifying therapy for PD. Current therapies are primarily based on a dopamine replacement strategy and effectively treat the motor features of PD but cannot prevent neurodegeneration. Researchers have found that Mucuna pruriens extract has suggestively ameliorated MPTP-induced neuroinflammation and restored the biochemical and behavioral abnormalities in PD mice [38]. The therapeutic effect of chlorogenic acid on MPTP-poisoned mice is also caused by its antioxidant and anti-inflammatory activity [39]. c-Abl is a nonreceptor tyrosine kinase implicated in oxidative stress response; previous study has found that the lack of c-Abl-induced antioxidant stress response is mediated by Nrf2 [40]. In our study, we found that STI 571 could prevent PHB2 tyrosine phosphorylation to improve mitophagy and reduce oxidative stress, which provided a basis for further testing STI 571 as a neuroprotective drug for Parkinson's disease. The c-Abl autophosphorylation at the Y245 site can promote kinase activity and can be used as a marker of c-Abl activation. The levels of unphosphorylated and phosphorylated c-Abl can be used to directly evaluate c-Abl activation [41]. However, c-Abl phosphorylation in vivo may occur in a sequential manner, with phosphorylation at Y412 occurring first, followed by Y245 phosphorylation [25]. Our experiment examined only the protein expression of p-c-Abl Y245, and whether p-c-Abl Y412 is involved in PHB2-mediated mitophagy had not been studied.

As a new mitophagy receptor, PHB2 plays an important role in antioxidative stress injury. In resting cells, PHB2 is stable on the mitochondrial inner membrane. However, PHB2 binds the autophagosomal membrane-associated protein LC3 through an LC3-interaction region (LIR) domain when mitochondrial depolarization and proteasome-dependent outer membrane rupture occur [22]. Upon mitochondrial membrane depolarization or misfolded protein aggregation, PHB2 depletion destabilizes PINK1 in the mitochondria, which blocks the mitochondrial recruitment of PRKN/Parkin, ubiquitin, and OPTN (optineurin), which leads to inhibition of mitophagy. In addition, overexpression of PHB2 directly induces PRKN recruitment to mitochondria [8]. However, the phosphorylation outcomes and regulatory mechanisms of PHB2 have not been reported in Parkinson's disease. In this study, we found that phosphorylation at the Y121 site of PHB2 reduced the binding of PHB2 to LC3 and decreased the level of mitophagy. Supplementation with PHB2 Y121 also did not ameliorate mitochondrial damage in a PD cell model with low PHB2 expression. Taken together, inhibition of PHB2 phosphorylation to enhance mitophagy may be a novel approach for the treatment of Parkinson's disease. Moreover, we found that STI 571 improved autophagy disorders by inhibiting PHB2 Y121 phosphorylation in the PD model. However, whether STI 571 can inhibit the phosphorylated protein expression of PHB2 Y121 is unclear in PD mice. Therefore, we need to further prepare phosphorylated antibodies at the PHB2 Y121 site by myself for further observation.

## 5. Conclusion

Our study found that phosphorylated c-Abl prevented the interaction of PHB2 with LC3 by increasing phosphorylation at the Y121 site of PHB2 and decreasing mitophagy and aggravated oxidative stress injury. STI 571 can inhibit the phosphorylation of c-Abl, enhance the interaction between PHB2 and LC3, and improve the behavioral defects of PD mice. The c-Abl-PHB2 signaling pathway provides a new pathway and target for the pathological mechanism of PD.

## Data Availability

The datasets generated and/or analyzed during the present study are available from the corresponding author upon reasonable request.

## Additional Points

*Materials and Correspondence*. The original contributions presented in the study are included in the article material; further inquiries can be directed to the corresponding authors.

## Supplementary Materials

Supplementary Materials Include Table S1 and Figure S1-S3. Supplementary Table 1: the base line data of rotarod test. Supplementary Figure S1: (a–c) the expression of p-c-Abl and PHB2 in the control shRNA and pEGFP-N1 groups compared with the control group. Supplementary Figure S2: (a–c) western blotting and quantitative analysis of PHB2 and LC3 expression in substantia nigra of mice in the sham group and control shRNA group. (d, e) The TH immunofluorescence (green fluorescence) in the substantia nigra compacta of the middle brain was analyzed statistically to determine the number of TH-positive neurons. Supplementary Figure S3: the quantitative analysis of base line data in rotarod test.
