# Supplementary material for: Nonreceptor Tyrosine Kinase c-Abl-Mediated PHB2 Phosphorylation Aggravates Mitophagy Disorder in Parkinson's Disease Model
Source: Oxid Med Cell Longev. 2022 Nov 9;2022:9233749. doi: 10.1155/2022/9233749 (PMC9668474; doi:10.1155/2022/9233749)
Supplement: Supplementary Materials — Include Table S1 and Figure S1-S3. Supplementary Table 1: the base line data of rotarod test. Supplementary Figure S1: (a–c) the expression of p-c-Abl and PHB2 in the control shRNA and pEGFP-N1 groups compared with the control group. Supplementary Figure S2: (a–c) western blotting and quantitative analysis of PHB2 and LC3 expression in substantia nigra of mice in the sham group and control shRNA group. (d, e) The TH immunofluorescence (green fluorescence) in the substantia nigra compacta of the middle brain was analyzed statistically to determine the number of TH-positive neurons. Supplementary Figure S3: the quantitative analysis of base line data in rotarod test. [file 9233749.f1.docx]

**Supplementary Materials**

**
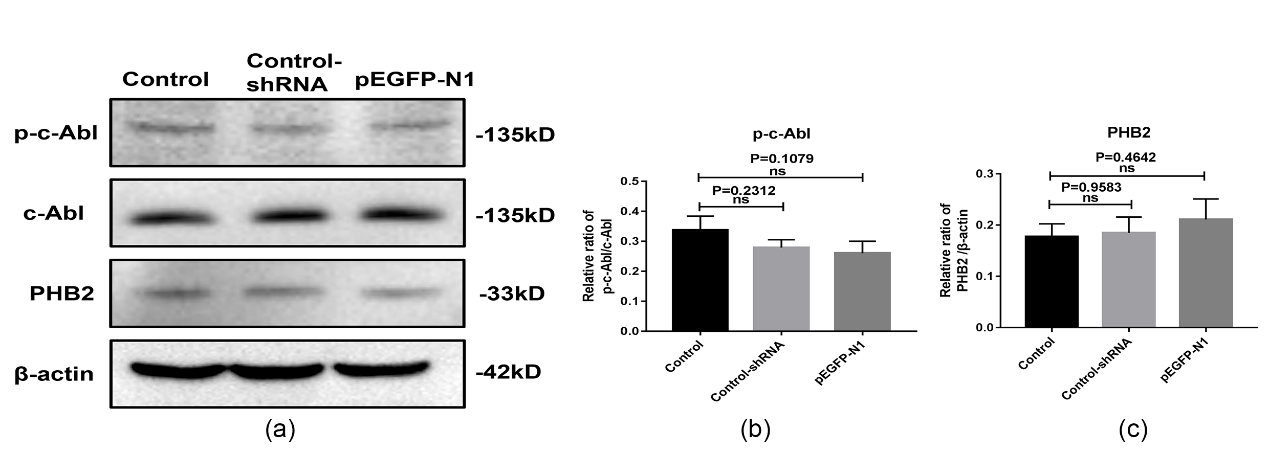
**

**Supplementary Figure S1: The expression of p-c-Abl and PHB2 in control shRNA and pEGFP-N1 groups was not significant when compared with the control group.**

**(a)**: Western blotting analysis of p-c-Abl, c-Abl and PHB2 expression in SH-SY5Y cells transfected with control shRNA (PHB2-shRNA vector) and pEGFP-N1 (PHB2 Y121D vector). **(b)-(c)**: p-c-Abl and PHB2 protein concentration changes by quantitative analysis.

**
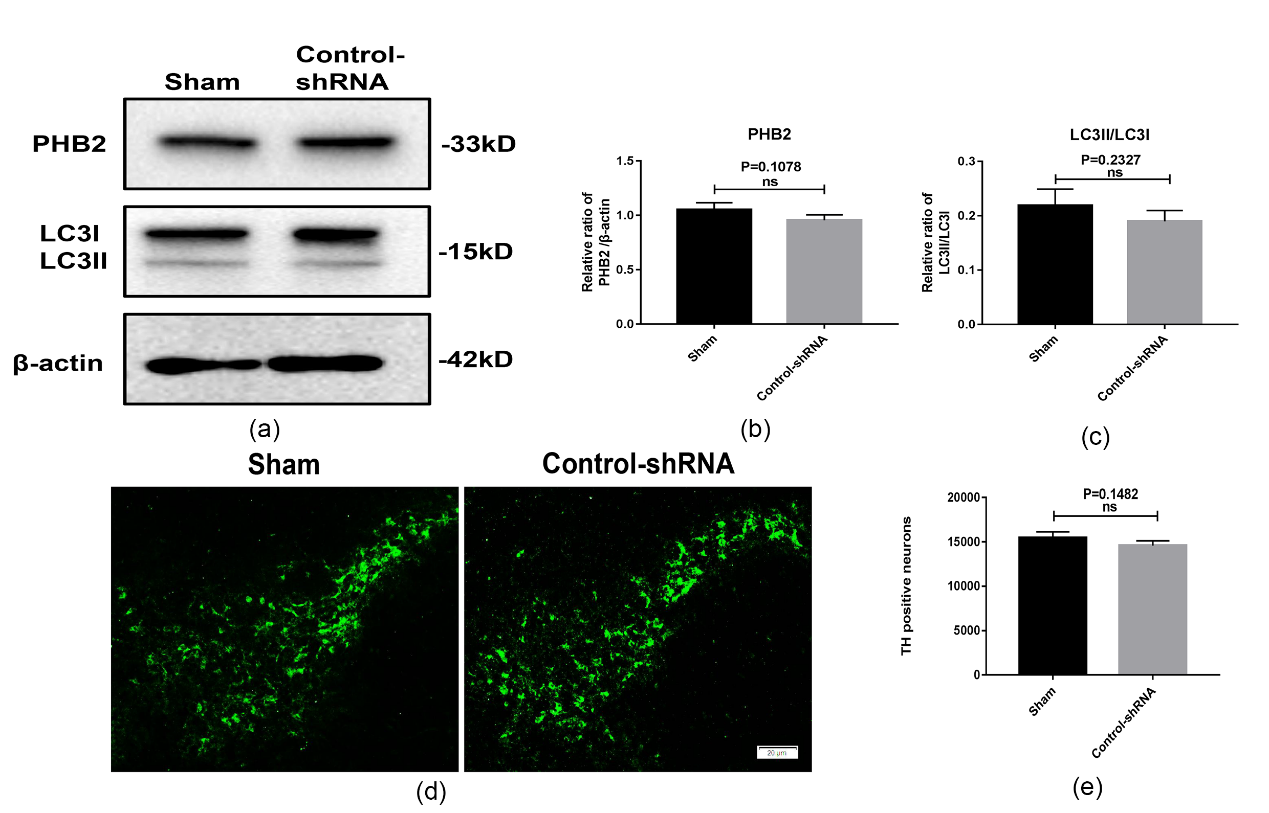
**

**Supplementary Figure S2:** **The expression of PHB2, LC3 and TH counts in Sham and control shRNA groups was not significant.**

**(a)**: Western blotting analysis of PHB2 and LC3 expression in substantia nigra of mice in sham group and control shRNA group. **(b)-(c)**: PHB2 and LC3 protein concentration changes by quantitative analysis. **(d)-(e)**: The TH immunofluorescence (green fluorescence) in the substantia nigra compacta of the middle brain was analyzed statistically to determine the number of TH-positive neurons. Bar=20 μm.

| Control  (n=5) | Subject ID | Speed | Duration(sec) |
| --- | --- | --- | --- |
|  | 1 | 4.0 to 40 RPM | 150 |
|  | 2 | 4.0 to 40 RPM | 127 |
|  | 3 | 4.0 to 40 RPM | 141 |
|  | 4 | 4.0 to 40 RPM | 156 |
|  | 5 | 4.0 to 40 RPM | 154 |
| MPTP  (n=5) | Subject ID | Speed | Duration(sec) |
|  | 1 | 4.0 to 40 RPM | 160 |
|  | 2 | 4.0 to 40 RPM | 142 |
|  | 3 | 4.0 to 40 RPM | 137 |
|  | 4 | 4.0 to 40 RPM | 139 |
|  | 5 | 4.0 to 40 RPM | 151 |
| STI 571  (n=5) | Subject ID | Speed | Duration(sec) |
|  | 1 | 4.0 to 40 RPM | 149 |
|  | 2 | 4.0 to 40 RPM | 151 |
|  | 3 | 4.0 to 40 RPM | 143 |
|  | 4 | 4.0 to 40 RPM | 139 |
|  | 5 | 4.0 to 40 RPM | 144 |
| MPTP+STI 571  (n=5) | Subject ID | Speed | Duration(sec) |
|  | 1 | 4.0 to 40 RPM | 144 |
|  | 2 | 4.0 to 40 RPM | 155 |
|  | 3 | 4.0 to 40 RPM | 136 |
|  | 4 | 4.0 to 40 RPM | 137 |
|  | 5 | 4.0 to 40 RPM | 142 |

**Supplementary Table S1: The base line data of rotarod test.**

**
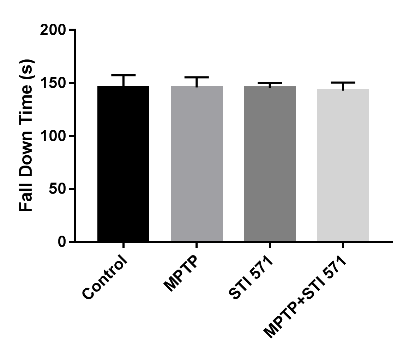
**

**Supplementary Figure S3:** **The quantitative analysis of base line data in rotarod test.**
